# Hypertrophy of the Prostate

**Published:** 1885-12

**Authors:** A. McNeil

**Affiliations:** San Francisco


### HYPERTROPHY OF THE PROSTATE.

PROFESSOR A. McNEIL, M. D., SAN FRANCISCO.

Dr. B. Ehrman's article interests me very much, and it recalled a case to my mind confirming his statements. While living in Indiana, passing a house, I was called in. The patient was a mulatto, fifty-seven years of age. I saw that he was suffering from general dropsy, and he informed me that he was also suffering from an enlarged prostate. He had been the servant of Dr. Ishem, of Louisville, Ky., and he and Professor Holland, of the University of Kentucky, had agreed in that diagnosis. I hastily considered that from the dropsy and the urinary symptoms, complicated by the enlarged prostate, I could not cure, so I did not make an examination myself nor did I test the urine.

He for some months had passed no urine, except by the catheter, which he had been taught to use. He had intense thirst, but drinking water caused distress and vomiting. He had a great deal of restlessness and slept but little. With no hope of giving him more than transitory relief, I gave him Arsen.$^{30}$ in water. In a short time there was marked relief. He soon began to pass part of his urine in the natural way, and before long his catheter was no longer necessary. The dropsy soon went, and the dyspnœa, which had tortured him, also, so that he frequently walked to my office, a distance of half a mile. I gave him Arsen. in increasing higher potencies as the action was exhausted, not giving him any medicine as long as improvement lasted. I kept him in sight for seven months, when he felt ready to go to work. During all of this time, after the first week, he had no necessity to use a catheter. I am fully aware that non-Hahnemannians will say that is "another high dilution lie," but I ask them to study up such cases of enlarged prostates carefully, according to the rules laid down by Hahnemann for the examination of the sick, and give the indicated remedy, no lower than the thirtieth, and as long as improvement continues give no medicine whatever. When improvement ceases, re-examine and give a dose of the similar remedy, let it be the first or another, and then report the results, giving all the symptoms, so that all may judge as to the homœopathicity of the remedy, to THE HOMŒOPATHIC PHYSICIAN. Be sure of the diagnosis.

I do not claim that all will be cured, but enough will to prove that a disease which sooner or later ends in death is amenable to homœopathic treatment.

